# Frontoparietal Tracts Linked to Lateralized Hand Preference and Manual Specialization

**DOI:** 10.1093/cercor/bhy040

**Published:** 2018-04-21

**Authors:** Henrietta Howells, Michel Thiebaut de Schotten, Flavio Dell’Acqua, Ahmad Beyh, Giuseppe Zappalà, Anoushka Leslie, Andrew Simmons, Declan G Murphy, Marco Catani

**Affiliations:** Natbrainlab, Sackler Institute for Translational Neurodevelopment, Forensic and Neurodevelopmental Sciences, Institute of Psychiatry, Psychology & Neuroscience, Denmark Hill, London, UK; Centre for Neuroimaging Sciences, Institute of Psychiatry, Psychology & Neuroscience, King’s College London, Denmark Hill, London, UK; Brain Connectivity and Behaviour Group, Sorbonne Universities, Hôpital de la Salpêtrière, Paris, France; Frontlab, Institut du Cerveau et de la Moelle épinière (ICM), UPMC UMRS 1127, Inserm U 1127, CNRS UMR 7225, Paris, France; Natbrainlab, Sackler Institute for Translational Neurodevelopment, Forensic and Neurodevelopmental Sciences, Institute of Psychiatry, Psychology & Neuroscience, Denmark Hill, London, UK; Centre for Neuroimaging Sciences, Institute of Psychiatry, Psychology & Neuroscience, King’s College London, Denmark Hill, London, UK; Natbrainlab, Sackler Institute for Translational Neurodevelopment, Forensic and Neurodevelopmental Sciences, Institute of Psychiatry, Psychology & Neuroscience, Denmark Hill, London, UK; Centre for Neuroimaging Sciences, Institute of Psychiatry, Psychology & Neuroscience, King’s College London, Denmark Hill, London, UK; Garibaldi Hospital, Piazza Santa Maria di Gesú, 5, Catania, Italy; Centre for Neuroimaging Sciences, Institute of Psychiatry, Psychology & Neuroscience, King’s College London, Denmark Hill, London, UK; Centre for Neuroimaging Sciences, Institute of Psychiatry, Psychology & Neuroscience, King’s College London, Denmark Hill, London, UK; Natbrainlab, Sackler Institute for Translational Neurodevelopment, Forensic and Neurodevelopmental Sciences, Institute of Psychiatry, Psychology & Neuroscience, Denmark Hill, London, UK; Natbrainlab, Sackler Institute for Translational Neurodevelopment, Forensic and Neurodevelopmental Sciences, Institute of Psychiatry, Psychology & Neuroscience, Denmark Hill, London, UK; Centre for Neuroimaging Sciences, Institute of Psychiatry, Psychology & Neuroscience, King’s College London, Denmark Hill, London, UK

**Keywords:** diffusion imaging tractography, handedness, hemispheric asymmetry, manual specialization, motor cognition, superior longitudinal fasciculus (SLF)

## Abstract

Humans show a preference for using the right hand over the left for tasks and activities of everyday life. While experimental work in non-human primates has identified the neural systems responsible for reaching and grasping, the neural basis of lateralized motor behavior in humans remains elusive. The advent of diffusion imaging tractography for studying connectional anatomy in the living human brain provides the possibility of understanding the relationship between hemispheric asymmetry, hand preference, and manual specialization. In this study, diffusion tractography was used to demonstrate an interaction between hand preference and the asymmetry of frontoparietal tracts, specifically the dorsal branch of the superior longitudinal fasciculus, responsible for visuospatial integration and motor planning. This is in contrast to the corticospinal tract and the superior cerebellar peduncle, for which asymmetry was not related to hand preference. Asymmetry of the dorsal frontoparietal tract was also highly correlated with the degree of lateralization in tasks requiring visuospatial integration and fine motor control. These results suggest a common anatomical substrate for hand preference and lateralized manual specialization in frontoparietal tracts important for visuomotor processing.

## Introduction

In human evolution, skilled use of the fingers has permitted a great leap forward in cultural and technological progress. The human hand has the special ability of being able to use the thumb in opposition to each finger, pad-to-pad, which permits a great variety of grips and finger manipulation for actions (Castiello 2005). Voluntary movement requires the capacity to generate independent finger movements, the transformation of sensory information into appropriate hand configurations and the interaction of perceptual and motor schemas (Jeannerod et al. 1995). Humans have exceptional abilities in relation to other primates in this regard, and a distinct feature of our species is the preferential use of the right hand over the left to perform complex motor tasks, a lateralized behavior that is referred to as handedness (Bishop 1990). Human handedness has been widely investigated using rating scales (Oldfield 1971; Dragovic 2004), behavioral tests (Tapley and Bryden 1985; Schmidt et al. 2000), and kinematics (Flowers 1975; Bagesteiro and Sainburg 2002), and more recenly using functional magnetic resonance imaging (fMRI) (Begliomini et al. 2008) and transcranial magnetic stimulation (TMS) (Sartori et al. 2013, 2014). However, its neuroanatomical basis in relation to cerebral connections remains largely unknown.

Historically, research on the anatomical correlates of human handedness has focused on cerebral structures involved in the final stages of fine motor command and coordination such as the cerebellum, the corticospinal tract, and the precentral gyrus. The cerebellum has been extensively studied in relation to hand movement, and despite a fixed ratio of neurons between the cerebellum and cerebral cortex across species (Herculano-Houzel 2012), there is a disproportionally larger volume of the dentate nucleus in humans as compared with other species (Matano and Hirasaki 1997). This has led to the hypothesis that human handedness may be related to an asymmetry of the dentate nucleus, although this is not supported by a recent volumetric MRI study performed in over 2000 subjects (Kavaklioglu et al. 2016). The bulk of the corticospinal tract originates in the motor cortex and activates moto-neurons in the contralateral spinal cord (Porter and Lemon 1993). A leftward asymmetry of the volume of these corticofugal tracts extending from the motor cortex (Nathan et al.1990; Rademacher et al. 2001; Thiebaut de Schotten, ffytche et al. 2011) and their pattern of decussation at the pyramidal level has consistently been reported (Kertesz and Geschwind 1971). However, these leftward anatomical asymmetries are equally observed in right- and left-handers, suggesting that hand preference may not be linked to corticospinal tract asymmetry (Kertesz and Geschwind 1971; Westerhausen et al. 2007).

In the cerebral hemispheres, the central sulcus divides the postcentral from precentral gyrus, that contain the majority of projections of the corticospinal tract. An effect of handedness on morphological aspects of the central sulcus has been reported in relation to its depth, which is deeper in the left hemisphere of right-handers as compared with left-handers (Amunts et al. 2000), and shape, in particular the hand-knob region is often located more dorsally in right-handers as compared with left-handers (Sun et al. 2012). Differences in central sulcus anatomy represent the most consistent finding in studies of human handedness, but these cannot be explained by morphological differences in the corticospinal tract (Kertesz and Geschwind 1971; Westerhausen et al. 2007). Other white matter pathways converging in the posterior frontal region may, therefore, be responsible for the reported morphological differences. In support of this hypothesis Büchel et al. (2004) reported higher fractional anisotropy in the white matter of the left precentral gyrus as compared with the right, in right-handers, and an opposite pattern in left-handers. The voxel-wise approach to the diffusion imaging data used by these authors was unable to identify specific tracts implicated in this asymmetry (Dell’Acqua and Catani 2012).

Tract-specific measurements have recently been studied with diffusion tractography for the short association U-fibers between the precentral and postcentral hand region (Catani, Dell’Acqua et al. 2012). In right-handers, the diffusion properties of the left U-fibers of the hand region are correlated with performance speed in a peg manipulation task (Thompson et al. 2017) and are significantly left-asymmetric in volume (Catani, Dell’Acqua et al. 2012). However, Magro et al. (2012) have shown that handedness again bears no relevance on the asymmetry of these tracts. In addition to short U-fibers, long association tracts connecting the frontal and parietal lobe have been extensively investigated in relation to reaching and grasping in nonhuman primates (Rizzolatti et al. 1998) and more recently in humans (Budisavljevic, Dell'Acqua et al. 2016). These tracts correspond to the three branches of the superior longitudinal fasciculus (SLF) (Petrides and Pandya 1984; Makris et al. 2005; Thiebaut de Schotten, Dell’Acqua et al. 2011) and are responsible for integrating visuospatial and somatosensory information to elaborate preparatory motor programs for prehension (Rizzolatti et al. 1998; Budisavljevic, Dell'Acqua et al. 2016; Parlatini et al. 2017). Asymmetry of SLF volume has been studied in relation to lateralization of visuospatial attention tasks and motor performance (Thiebaut de Schotten, Dell’Acqua et al. 2011; Budisavljevic et al. 2016). In a group of right-handers, the degree of rightward asymmetry of the second branch of the SLF was highly correlated with leftward deviation in a line-bisection task as well as a faster response with the left hand compared with right hand on the Posner paradigm. Asymmetry of the SLF in right-handers has been also shown to correlate with faster reaching acceleration with the right hand (Budisavljevic, Dell'Acqua et al. 2016). These results suggest that SLF asymmetries may be an important anatomical correlate of hand preference and manual specialization. A recent tractography study has reported no volume difference in the three SLF branches between right- and left-handers, although the small number of participants may have affected statistical power to detect between-group differences (Cazzoli and Chechlacz 2017).

In our study, 51 healthy participants were recruited and advanced diffusion tractography used to dissect two groups of motor white matter tracts: (1) the three association frontoparietal branches of the SLF involved in multisensory integration and motor planning; (2) the projections of the corticospinal tract and superior cerebellar peduncle involved in conveying motor commands and coordination. First, we evaluated whether structural differences in these tracts existed between right- and left-handers. Second, behavioral tasks involving either object manipulation or alternating finger movements were used to understand the mechanisms underlying a possible link between structural asymmetry, hand preference, and manual specialization.

## Materials and Methods

### Participants

In total, 51 healthy adults participated in the study (23 males, mean age 27 ± 4 years). Written and informed consent to participate in this research was obtained. The Psychiatric, Nursing and Midwifery subcommittee of the College Research Ethics Committee at King’s College London approved the study. Exclusion criteria included preterm or difficult birth, a history of neurological or psychiatric disease, and chronic diseases that involved bones and connective tissue (Satz 1973). Participants with a history of fractures involving bones of the upper limbs that required restricted healing for longer than 6 months were also excluded.

### Classifying Hand Preference

Handedness can be defined as an overall preference to use one hand over the other for familiar activities such as writing or object manipulation. At the population level, right-handers are most commonly strongly right-handed across tasks, whereas left-handers have a more heterogeneous degree of manual lateralization and for this reason they are often referred to as “non-right-handers” in the literature. In this study we kept to the description of left-handers for ease of reading. Participants were screened for their handedness profile using the Edinburgh Handedness Inventory (EHI) (Oldfield 1971). The EHI scores of the 30 right-handers and 21 left-handers are reported in Supplementary Figure S1. We defined the handedness groups using only the choice of hand for the unimanual tasks but excluded the choice of hand for scissors, which is often enforced to the right due to tool design (Bryden 1977; McFarland and Anderson 1980; Veale 2014).

### Classifying Unimanual Skill or Performance

Manual specialization differs from handedness in that it refers to the ability, rather than the preference, of one hand over the other to perform unimanual tasks (Supplementary Fig. S1). Specific tests can be used to assess unimanual performance and determine asymmetry between the two hands as a continuous variable. To test for visuomotor unimanual skill, the Grooved Pegboard was administered to a subset of 31 participants (13 left-handers, 15 males) (Lafayette Instruments). This task requires reaching and grasping movements, and finger manipulation. The pegboard consists of a 5 × 5 inch^2^ metal surface containing a matrix of evenly spaced keyhole-shaped holes in different orientations. Participants were required to place 3 mm diameter pegs held in a receptacle on the board, into the holes, as fast as they could with the dominant and then the nondominant hand, and again for a second trial (the place condition). After each place condition, participants were timed in the speed of replacing the pegs as quickly as possible from the board into the receptacle (the remove condition). The Grooved Pegboard is a goal-directed task, necessitating intact motor programming to manipulate pegs and cooperation of the arm, wrist and at least 2 fingers (Klôve 1963; Bryden and Roy 2005). The task requires both an impulse (planning) phase, where the hand approaches the target, and an online control phase, where adjustments (error detection and correction) are made using visual, tactile and proprioceptive feedback. A high level of visual control is necessary for the place condition due to the alternately oriented keyholes, whilst removing the pegs requires less visual feedback (Albuquerque et al. 2017). It has been calculated that the place condition has a Fitts’ index of difficulty (ID) of 9 bits whereas the remove condition an ID of 1.5 bits (Fitts 1954, Bryden and Roy 1999). The ID of the place condition is likely to represent an underestimation of the actual difficulty due to the complexity of the keyhole component that is not taken into account in the calculation. The speed at which the subject performed each condition was recorded in seconds. The order in which hands are tested can affect performance (Schmidt et al. 2000), therefore participants were tested using first the dominant hand then the nondominant hand (trial 1) and the same again (trial 2). We used the scores from the second trial, using performance difference between the first and second trial as a covariate, to attempt to factor in potential intermanual transfer of training.

Participants were also tested on their speed of unimanual finger movement with each hand, using an index finger tapping and a coordinated alternating (thumb-middle-second-ring-repeat) finger tapping test. The task was designed using Superlab 5 (Cedrus, USA) and a Superlab RB-830 response pad. Subjects were asked not to look at their fingers during the task, but to keep focused on the “Go” sign on the computer screen ahead for the length of the trial. They were also asked to keep the palm down on the pad so to not use the proximal musculature in the task. A practice trial was given to participants until they were deemed to be able to perform the task without making errors. Participants started with their dominant hand for 5 trials of 10 s and the number of taps was recorded. Errors in performance were also recorded but were not sufficient to considerably alter performance speed. Both tapping tests assessed motor co-ordination of the index finger only (single tapping) or four digits (alternating tapping), and therefore rely on tactile somatosensory information without any visual feedback.

Lateralization indices (LI) were produced for the behavioral tasks to create a spectrum of relative hand skill. For the pegboard, the LI was calculated using (left hand – right hand)/(left hand + right hand), using time to completion as the measure (lower speed means better performance). For the finger tapping tasks, the LI was calculated using (right hand – left hand)/(right hand + left hand), using the mean number of taps across 5 trials with the relevant hand as the measure (higher number of taps means best performance). These provided a spectrum of relative performance between the hands for each task, between −1 (best performance with left than right hand) and 1 (best performance with right than left hand). The LI accounted for some possible confounding variables such as muscle size, length of peripheral nerves and attention.

### Image Acquisition and Processing

Diffusion datasets were acquired in all 51 participants, on a 3 T General Electric Signa HDx TwinSpeed system MRI scanner with an 8 channel head coil. For diffusion weighting, a spin-echo single-shot echo planar imaging (EPI) sequence was used with a voxel size of 2.4 × 2.4 × 2.4 mm^3^, matrix of 128 × 128 and a field of view of 307 × 307 mm^2^. Overall, 60 contiguous near-axial slices were acquired with an echo time of 93.4 ms along 60 diffusion-weighted directions. A *b*-value of 3000 s/mm^3^ was used, and 7 volumes with no diffusion weighting were also collected. Data was acquired using an ASSET factor of 2 and cardiac gating with an effective TR of 20/30 R–R intervals.

Data was corrected for head motion and eddy current distortion using ExploreDTI (Leemans et al. 2009). For each subject, the *b*-matrix was then reoriented to provide a more accurate estimate of diffusion tensor orientations (Leemans and Jones 2009). Spherical deconvolution was calculated applying the damped version of the Richardson–Lucy algorithm with a fiber response parameter of *α* = 1.5, 200 algorithm iterations, an ALFA value of 2, threshold parameters of 0.04, and geometrical regularization parameters of 8 (Dell’Acqua et al. 2010). Fiber orientation estimates were obtained by selecting the orientation corresponding to the peaks (local maxima) of the fiber orientation distribution (FOD) profiles. To exclude spurious local maxima, we applied both an absolute (value 0.06) and a relative (value 5) threshold on the FOD amplitude (Dell’Acqua et al. 2013).

Whole brain tractography was run on the diffusion datasets using a step size of 1 mm with a limit set to display only streamlines between 15 and 400 mm in length (mean number of streamlines in a whole brain tractogram was 69 248.9 ± 9517). The Euler algorithm was used to follow the orientation vector of least curvature (angle threshold of 60°), thus, allowing to track through crossing (Catani, Bodi et al. 2012; Dell’Acqua et al. 2013). All spherical deconvolution and tractography analysis was performed using StarTrack software (www.natbrainlab.com).

### Virtual Dissections for White Matter Tracts

Manually guided dissections were performed in each hemisphere using a region-of-interest (ROI) constrained approach in TrackVis software (Wang et al. 2007). The dissector (H.H.) was trained by expert tractographers (M.C. and M.T.S.) using multiple inclusion and exclusion ROIs detailed in previous papers (Thiebaut de Schotten, Dell’Acqua et al. 2011; Thiebaut de Schotten, ffytche et al. 2011). Large ROIs were symmetrically drawn in each hemisphere to incorporate the full white matter region visible in the relevant plane. The general approach adopted here is outlined in Catani and Thiebaut de Schotten (2008) and uses ROIs delineated around the stems of each tract and avoids using cortical ROIs in order to retain the individual anatomy of each brain. The following tracts were included in the analysis, and images of the ROIs used for dissection are shown in Supplementary Figure S2.

#### Frontoparietal Association Pathways of the SLF

**Figure 1.**
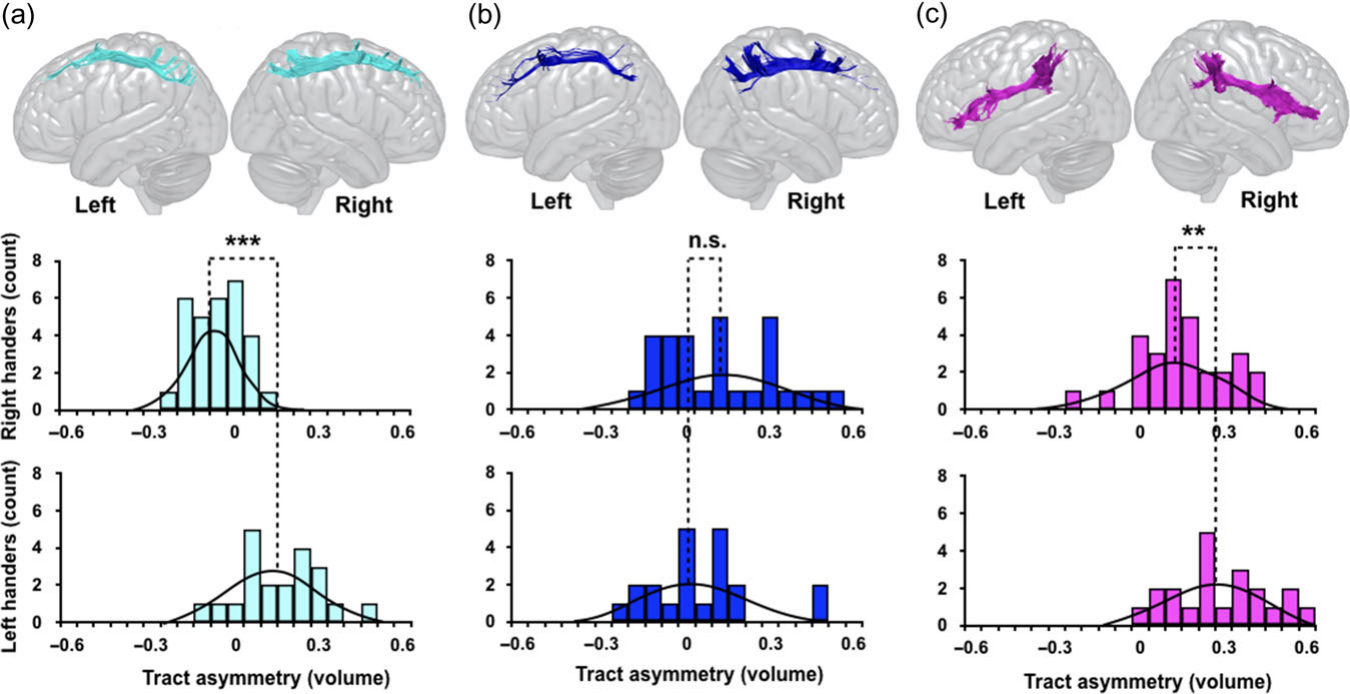
The distribution of hemispheric asymmetry of the (a) SLF I, (b) SLF II, and (c) SLF III in right-handers (top row) and left-handers (bottom row). A negative laterality index (LI) reflects larger tract volume in the left hemisphere than the right. Statistical analysis was performed using paired-*t*-test between right- and left-handers (***P < 0.001, **P < 0.005, both values survive Bonferroni corrections). The tractography images of the three branches of the SLF are from a left-hander participant.

In humans, the most dorsal subcomponent, the SLF I, connects the superior parietal lobule and the superior frontal gyrus, as shown in Figure 1a and Supplementary Figure S3. It runs parallel but dorsal to the cingulum, from which is separated by the cingulate sulcus and medial callosal fibers. To dissect this branch, an anterior ROI was placed in the coronal plane within the superior frontal gyrus, extending within the white matter until the cingulate sulcus. A second posterior ROI was specified in the parietal lobe in the coronal plane just behind the postcentral gyrus. A ROI was also drawn to exclude fibers extending into the temporal and occipital lobe. This ROI was used for all three branches of the SLF.

The SLF II is shown in Figure 1b and Supplementary Figure S3. It runs below the SLF I but through the middle frontal gyrus, connecting dorsal premotor and prefrontal cortices with caudal portions of the inferior parietal lobule. For tractography dissection, an anterior ROI was drawn in the coronal plane to delineate the caudal middle frontal gyrus, whilst the same parietal ROI delineated for the SLF I was used to visualize the posterior extent of the SLF II.

The third subcomponent, the SLF III is the most ventral branch as shown in Figure 1c and Supplementary Figure S3. It connects the inferior frontal gyrus with the intraparietal sulcus and rostral inferior parietal lobule. The SLF III was dissected using one anterior ROI in the coronal plane anterior to the ventral precentral gyrus and another inclusion ROI in the parietal lobe.

#### Projection Pathways of the Corticospinal Tract and Superior Cerebellar Peduncle

**Figure 2.**
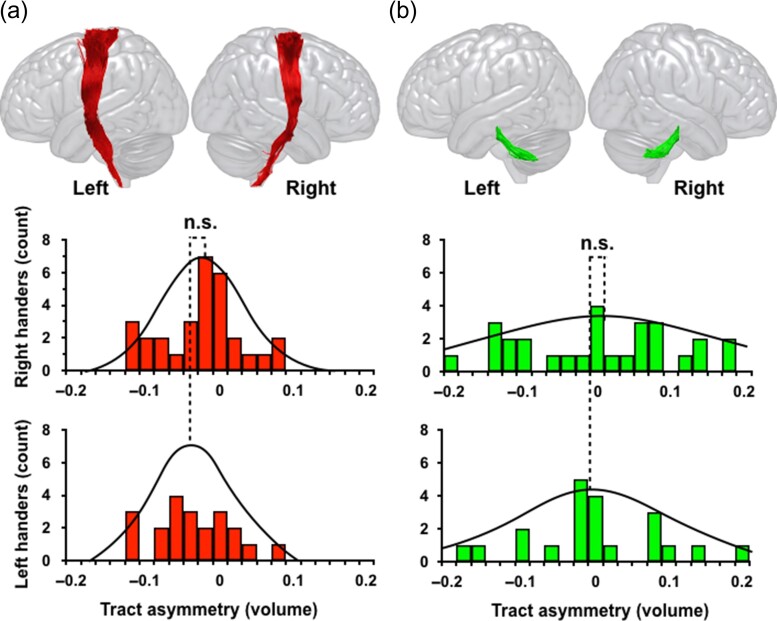
The distribution of hemispheric asymmetry of the (a) corticospinal tract and (b) superior cerebellar peduncle in right-handers (top row) and left-handers (bottom row). A negative laterality index reflects larger tract volume in the left hemisphere than the right. The tractography images of the corticospinal tract and superior cerebellar peduncle are from a left-hander participant.

The corticospinal tract, shown in Figure 2a, originates mainly from the precentral gyrus, runs through the cerebral peduncles and connects to the spinal cord. This pathway is traced using one inclusion ROI within the white matter of the precentral gyrus and another ROI in the anterior portion of the ipsilateral cerebral peduncles. A large mid-sagittal exclusion ROI was used to eliminate artefactual streamlines (Thiebaut de Schotten, Dell’Acqua et al. 2011).

The superior cerebellar peduncle originates in the deep cerebellar nuclei and is shown in Figure 2b. The first ROI was placed around the white matter surrounding the dentate nucleus. The second ROI was placed in the central part of the superior cerebellar peduncle (Catani et al. 2008). Only streamlines traveling between the 2 ROIs were included in the analysis to avoid artefactual contamination from thalamic connections.

### Statistical Analysis

The total volume of space occupied by the streamlines of each tract was extracted for each hemisphere (measured in mm^3^) and used as the tract volume measurement. As for the behavioral tasks, a lateralization index was calculated using the following formula: LI = (right hemisphere tract volume – left hemisphere tract volume)/(right hemisphere tract volume + left hemisphere tract volume). A value at 0 shows a bilateral distribution, while negative values indicate a leftward asymmetry (greater tract volume in the left hemisphere), and conversely a rightward asymmetry when a positive value (Catani et al. 2007).

Statistical comparisons were performed using SPSS software (SPSS, Chicago). Shapiro–Wilk tests were used to verify normality of anatomical and behavioral variables. A repeated measures ANOVA was used to assess the interaction between handedness (right-handers vs. left-handers), hemisphere (right hemisphere vs. left hemisphere) and volume of each tract after covarying for sex. A repeated measures ANOVA was also used to evaluate the interaction between handedness, task condition (place vs. remove), hand used (left vs. right) and behavioral performance on the pegboard task after covarying for sex. A similar analysis was performed for the single finger and multifinger tapping tasks. Post hoc tests (ANOVA) were performed to examine group differences in single tract volumes, tract asymmetries, and behavioral performances on the Pegboard task and finger tapping tasks. Effect size was calculated using partial eta squared (*η_p_*^2^, Cohen 1973). Hierarchical logistic regression was performed to assess the effect of tract asymmetry on handedness. Linear regression was performed to evaluate the association between volume asymmetry of all tracts and variance in lateralized hand performance on the 2 behavioral tasks. Significance was set at *P* value < 0.05. One-sample *t*-tests were used to assess statistically significant tract asymmetries in each handedness group.

Partial Pearson product–moment correlations were used to show the patterns of association between structural and behavioral asymmetry including sex as a group regressor of no-interest (and practice effects for Grooved Pegboard). These were also used to assess relationships between tracts within each hemisphere and behavioral performance. Comparison of the correlations from the dependent samples was conducted using a Steiger’s *Z* (Steiger 1980) and effect size was calculated using Cohen’s *q* (Cohen 1988).

## Results

### Effect of Handedness on Tract Volume and Asymmetry

A repeated measures ANOVA showed a significant 3-way interaction between hemisphere, handedness, and tract volume (*F*[5,45] = 10.5, *P* < 0.001, *η_p_*^2^ = 0.5), which was significant only for the SLF I (*F*[5,45] = 41.04, *P* < 0.001, *η_p_*^2^ = 0.5) and SLF III (*F*[5,45] = 4.67, *P* = 0.036, *η_p_*^2^ = 0.09) as demonstrated by the univariate test. A one way ANOVA showed that compared with right-handers, left-handers had significantly larger volume of the SLF I (*F*[1,49] = 5.23, *P* = 0.03, *η_p_*^2^ = 0.6) in the right hemisphere (Table 1).

**Table 1:** Tract volume and asymmetry in the right- and left-handed group

	Left hemisphere (mm^3^)		Right hemisphere (mm^3^)		Asymmetry (LI)	
	Right-handers	Left-handers	Right-handers	Left-handers	Right-handers	Left-handers
SLF I	21.5 (8.2)	17.8 (5.9)	18.3 (7.8)	22.7 (5.3)**	−0.1 (0.08)	0.13 (0.14)***
SLF II	15.6 (6.7)	15.3 (8.9)	18.8 (8.0)	19.5 (7.1)	0.09 (0.19)	0.02 (0.18)
SLF III	16.5 (5.4)	14.5 (6.0)	21.7 (8.5)	23.6 (6.9)	0.12 (0.15)	0.26 (0.16)*
CST	20.2 (3.7)	20.0 (4.3)	18.9 (3.7)	18.1 (3.7)	−0.03 (0.05)	−0.05 (0.05)
SCP	3.0 (0.8)	3.4 (0.6)*	3.1 (0.7)	3.4 (0.68)	0.01 (0.13)	−0.01 (0.09)

Note: Values are mean scores shown with standard deviation. LI, lateralization index (negative values indicated leftward asymmetry); SLF, superior longitudinal fasciculus; CST, corticospinal tract; SCP, superior cerebellar peduncle. *P < 0.005 that survives Bonferroni correction for multiple comparisons. Asterisks indicate statistically significant differences between left- and right-handers that survive Bonferroni correction for multiple comparisons (*P < 0.05, **P < 0.005,***P < 0.001).

A binomial regression was performed to ascertain the relationship between tract asymmetry measured by the LI of each tract and handedness. The logistic regression model was statistically significant *χ*^2^(5) = 42.328 (*P* < 0.001) and explained 76% (Nagelkerke *R*^2^) of the variance in handedness, and correctly classified 84.3% of cases. No tract LIs were significantly correlated with another as evaluated by the models’ correlation matrix. Of the 5 variables, only the LI of the SLF I was significantly associated with handedness (*P* = 0.003; see Supplementary Table S1), although SLF II tract asymmetry approached significance (*P* = 0.064).

Post hoc tests showed a significant leftward LI for the SLF I in right-handers (*t*[29] = −5.62, *P* < 0.001) and rightward LI in left-handers (*t*[20] = 4.33, *P* < 0.001) (Table 1) with a statistically significant difference (*P* < 0.001) between groups (Fig. 1). There was a trend of rightward LI of the SLF II in the right-handers (*t*[29] = 2.44, *P* = 0.02) but not the left-handers (*t*[20] = 0.46, *P* = 0.6) (Table 1) with no statistically significant difference between groups (Fig. 1). A statistically significant rightward LI for the SLF III was observed for both right-handers (*t*[29] = 4.34, *P* < 0.001) and left-handers (*t*[20] = 7.10, *P* < 0.001) (Table 1) with a statistically significant difference between groups (*P* = 0.004) (Fig. 1). Of the projection tracts, the corticospinal tract was left-asymmetric in both the right-handers (*t*[29] = −3.40, *P* = 0.002) and left-handers (*t*[20] = −4.1, *P* = 0.001) (Table 1) without statistically significant differences between groups (Fig. 2). The superior cerebellar peduncle was bilateral in both right-handers (*t*[29] = 0.91, *P* = 0.4) and left-handers (*t*[20] =−0.40, *P* = 0.6) (Table 1 and Fig. 2).

### Manual Specialization on the Grooved Pegboard

We assessed the extent of manual specialization (better speed to completion with one hand compared with the other) on the Grooved Pegboard under the place and remove conditions and results are presented in Table 2.

**Table 2:** Scores on behavioral tasks in the right- and left-handers

	Left handers (*n* = 13)				Right handers (*n* = 18)			
	Left hand	Right hand	LI	Degree	Left hand	Right hand	LI	Degree
Peg place	47.8 (4.6)**	52.5 (7.9)	−4.3 (6.2)***	5.8 (4.8)	53.1 (6.7)	49.1 (6.3)	3.9 (5.2)	5.6 (3.1)
Peg remove	15.8 (1.7)**	16.5 (2.0)	−2.3 (4.3)***	3.6 (3.1)	17.9 (2.2)	17.4 (2.1)	1.4 (2.9)	2.3 (2.2)
Multifinger tapping	49.2 (10.9)	49.4 (10.3)	−0.1 (4.0)*	2.6 (2.9)	40.2 (9.2)	43.5 (11.8)	3.4 (4.8)	4.8 (3.3)
Index tapping	54.2 (10.7)	52.8 (7.1)	−0.7 (5.1)*	4.5 (2.1)	51.5 (5.7)	55.5 (5.5)	4.2 (3.9)	5.0 (2.8)

Note: Values are mean scores shown with standard deviation. Pegboard values are average time (seconds) to completion therefore lower values indicate better performance, whereas finger tapping values are number of taps within 10 s over 5 trials and therefore higher values indicate better performance. Degree indicates the extent of asymmetry, without taking into account the directionality. Asterisks indicate statistically significant differences between left- and right-handers that survive Bonferroni correction for multiple comparisons (*P < 0.05, **P < 0.005, ***P < 0.001).

Using a repeated measures analysis, a significant interaction between hand used, condition and handedness was observed after covarying for sex (*F*[1,28] = 11.6, *P* = 0.002, *η_p_*^2^ = 0.3). A 2-way ANOVA between groups (left- or right-handers) and condition (place or remove) showed a statistically significant effect of handedness but not condition on performance with the left hand (*F*[1,28] = 10.5, *P* < 0.005, *η_p_*^2^ = 0.3) but not for the right hand (*F*[1,28] = 0.44, *P* = 0.5, *η_p_*^2^ = 0.01). Left-handers performed better than right-handers on both conditions with their left hand.

A 2-way ANOVA between groups (left- or right-handers) and condition (place or remove) showed a statistically significant effect of handedness, but not condition, on LI of performance (*F*[1,28] = 17.5, *P* < 0.001, *η_p_*^2^ = 0.4).

Finally, the lateralization indices were transformed into absolute values to assess whether the degree of manual specialization was different between left- and right-handers irrespective of the direction of lateralization. A 2-way ANOVA using group × condition showed no significant effect of handedness or condition on the degree of manual specialization (*F*[1,28] = 0.58, *P* = 0.45).

### Relationship Between Manual Specialization on the Pegboard Task, Tract Volume and Tract Asymmetry

We used linear regressions to separately assess the extent to which volume asymmetry of all tracts were associated with lateralized hand performance on the place and remove condition, covarying for sex. On both models for the place and remove conditions there was homoscedasticity and independence of residuals as assessed by a Durbin–Watson statistic (place task: 1.729, remove task: 2.105). There was no effect of colinearity as assessed by the variance inflation factor (between 1 and 1.6). There was a statistically significant relationship between tract asymmetry and lateralized hand performance on the place task (*F*[6,24] = 3.6, *P* = 0.011), with SLF I tract asymmetry being the only significant predictor variable (*t*[6,24] = −3.71, *P* < 0.001). The associations were independent from the order of entry, as the SLF I remained a significant predictor even when variance of other tracts was taken into account (Supplementary Table S2).

A linear regression showed tract asymmetry approached significance as a predictor of lateralized hand performance on the remove task (*F*[6,24] = 2.36, *P* = 0.06).

**Figure 3.**
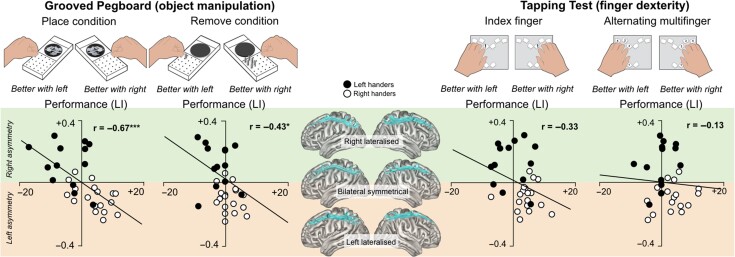
Scatter plots of the correlation between volume asymmetry measured by the lateralization index (LI) of the SLF I and behavioral lateralization on the Grooved Pegboard and finger tapping. Asterisks indicate statistically significant correlations (***P < 0.001, *P < 0.05). The tractography reconstructions of 3 subjects with different degrees of SLF asymmetry are shown in the middle panel.

Figure 3 shows the correlation analysis between SLF I asymmetry and lateralized hand performance for both place and remove conditions (Supplementary Table S3). A highly significant negative correlation was observed for the place (*r* = −0.6, *P* < 0.001) condition (Fig. 3a), indicating that participants that performed better with the left hand had larger volume of the right SLF I (compared with the left SLF I), while those who performed better with the right hand had larger volume of the left SLF I (compared with the right SLF I). A similar negative correlation, although statistically weaker, was also observed for the remove condition (Fig. 3b; *r* = −0.43, *P* = 0.01). The Steiger’s *Z* test showed that the partial correlations between hemispheric asymmetry of the SLF I and performance were not statistically different between the place and remove conditions (*Z*_H_ = −1.27, *P* = 0.205, Cohen’s *q* = 0.2). The strength of the correlation only remained significant for the place condition after covarying for left hand performance (*r* = −0.61, *P* < 0.001 to *r* = −0.5, *P* < 0.005), practice effects, and sex (*r* = −0.66, *P* < 0.001).

When the analysis was repeated for each handedness group separately, the relationship between volume asymmetry of the SLF I and lateralization of performance in the pegboard test was significant only for the right-handers (*r* = −0.542, *P* < 0.05) in the place condition with a trend towards significance also for the left-handers (*r* = −0.545, *P* = 0.06).

### Relationship Between Manual Specialization on the Finger Tapping Task and Tract Asymmetry

Performance on the index and multifinger tapping tasks, which required tactile and proprioceptive but no visual feedback and motor sequencing of only the distal musculature, are reported in Table 2. A repeated measures analysis of variance was performed, covarying for sex, that showed no statistically significant interaction between hand used (left or right), condition (single index vs. multifinger tapping), and handedness (*F*[1,28] = 1.06, *P* = 0.31).

An ANOVA between groups (left- or right-handers) and condition (index or multifinger tapping) showed no significant effect of condition (*F*(1,28) = 0.41, *P* = 0.52, ηp_2_= 0.05), but a significant effect of handedness (*F*(1,28) = 16.14, *P* < 0.001, ηp_2_ = 0.4) on LI of performance. Between-group differences were statistically significant for both index finger tapping (*F*[1,29] = 8.95, *P* = 0.006) and multifinger tapping (*F*[1,29] = 4.52, *P* = 0.042). The lateralization index was significantly right-lateralized on both index finger tapping (*t*[17] = 4.49, *P* < 0.001) and multifinger tapping (*t*[17] = 3.04, *P* < 0.005) in the right-handers, but not in the left-handers (index tapping task, *t*[12] = −0.478, *P* = 0.6; multifinger, *t*[12] = −0.039, *P* = 0.1).

When linear regression analysis was used to assess whether tract volume asymmetry was associated with lateralized hand performance, and the model was not significant for either index (*F*[6,24] = 1.19, *P* = 0.3) or multifinger (*F*[6,24] = 0.59, *P* = 0.7) tapping tasks, indicating that neither tract asymmetry of the association nor projection tracts was associated with lateralized performance on these tasks. On both these predictor models there was homoscedasticity and independence of the residuals (Durbin–Watson statistic; index tapping: 1.9; multifinger tapping: 2.0). Figure 3 shows that SLF I asymmetry was not significantly correlated with lateralized performance on both index finger (r = −0.33, *P* = 0.07) and multifinger tapping (r = −0.13, *P* = 0.49).

## Discussion

In this study, we used diffusion tractography to evaluate differences in tract asymmetry between right- and left-handers, and to assess whether this was associated with manual specialization as tested across a range of motor tasks. Our results indicate that although clear hemispheric asymmetries exist for several association and projection tracts, an effect of handedness was observed only on asymmetry of the volume of the most dorsal branch of the three frontoparietal association tracts (SLF I). Furthermore, lateralized motor performance between the hands on a unimanual peg placing task requiring a high degree of visual and somatosensory information was associated with SLF I asymmetry. Our findings may indicate an effect of handedness on the asymmetry of frontoparietal tracts involved in multisensory integration and planning of voluntary movement but not on the asymmetry of projection tracts involved in direct motor command.

### Frontoparietal Connections for Reaching and Grasping

Although our results are the first report of an association between structural asymmetry of the SLF I and hand preference, the role of frontoparietal tracts in supporting computations necessary for translating goal-directed action into movement has been established both in animals (Turella and Lingnau 2014) and more recently in humans (Budisavljevic, Dell'Acqua et al. 2016). In the parietal lobe, the SLF I connects to a dorsal region composed of several functional areas important for visuomotor integration (Gallivan et al. 2013), motor learning (Wenderoth et al. 2004), directing spatial attention (Corbetta et al. 1995), spatial perception (Weiss et al. 2003), memory (Zago and Tzourio-Mazoyer 2002), and mental rotation (Vingerhoets et al. 2002). In particular, the posterior superior parietal areas receive visual information from the superior colliculus (Goldberg and Wurtz 1972; Rushworth et al. 2005) and occipital cortex (Mishkin and Ungerleider 1982) for visually guided reaching movements (Battaglia-Mayer and Caminiti 2002) and are considered the termination of the dorsal visual stream for action (Goodale and Milner 1992). The more anterior parietal areas receive somatosensory information important for spatial representation of body parts such as the fingers (Jones et al. 1978; Bédard and Sanes 2009; Mars et al. 2011). Sensory signals from both visual (spatial) and tactile modalities are therefore combined in the superior parietal region (Catani et al. 2017) to form multiple representations of space, coding the spatial location of goals for movement, and integrating different components of arm transport, coordination, and grasping (Mountcastle et al. 1975; Andersen et al. 1997; Le et al. 2017). The superior parietal lobule thus plays an important role in comparing anticipated and actual sensory feedback for the correct execution of goal-directed actions (Wolpert et al. 1998; Grafton 2010; Shadmehr et al. 2010). In the frontal lobe, the SLF I projects to the superior frontal gyrus where different functional areas have been identified. On the medial aspect, the supplementary motor areas (SMA/pre-SMA) (Luppino et al. 1993) are important in motor sequencing (Tanji and Shima 1994), whereas dorsal and lateral premotor cortex is involved in reaching and grasping (Davare et al. 2006; Begliomini et al. 2007; 2015), working memory (Boisgueheneuc et al. 2006) and attention (Miller and Cohen 2001). Similarly to the parietal lobe, a spatial-to-effector gradient has been described also in the frontal lobe, however occuring in a reverse order (i.e., rostral-to-caudal) (Beurze et al. 2007, 2009; Stark and Zohary 2008). By linking the dorsal frontal and parietal regions, the SLF I contributes to transforming object affordances into specific motor schemata (Jeannerod 1994; Borra et al. 2017; Vesia et al. 2017) by programming proximal musculature (Vesia et al. 2010), selecting wrist posture (Monaco et al. 2011) and facilitating online control of grasping (Grol et al. 2007) during goal-directed actions.

A recent tractography study has identified a link between variability in the structural anatomy of the SLF and performance in upper limb visuomotor control (Budisavljevic, Dell’Acqua et al. 2016). These authors reported, in a group of right-handers, a moderate left hemisphere structural asymmetry of the SLF I and an association between specific kinematic features of the right hand and volume of the SLF II and SLF III in the right and left hemispheres separately. Our results are in line with these findings (Supplementary Table S3) but also indicate a prominent association between SLF I, handedness and manual specialization. Differences between Budisavljevic, Dell’Acqua et al. (2016) and our results may be related to the behavioral tasks used in the 2 studies. The pegboard test provides an overall measure of task performance that is affected by sustained attention and fatigue, especially on performance with the nondominant hand (Strenge et al. 2002). It is also based on visual search that has been linked to the activation of a dorsal frontoparietal network connected by the SLF I (Parlatini et al. 2017). Kinematic analysis, on the other hand, permits a more detailed breakdown of different aspects related to hand control, which may not be concordant with manual preference and may have no relation to pegboard performance (Sainburg 2002; Nelson et al. 2017). Taken together, these 2 studies indicate that while SLF I anatomy may represent the most significant tract associated with handedness and manual specialization, the SLF II and III may be important for other aspects of upper limb control, particularly in the absence of visual information (Fornia et al. 2016). TMS studies have demonstrated that activity in the ventral frontoparietal circuit precedes that in the dorsal frontoparietal circuit during grasping, suggesting a hierarchical organization between the two pathways reflecting different levels of abstraction: composing an object-based motor plan and then using this structure appropriately in space (Davare et al. 2006; Taubert et al. 2010; Verhagen et al. 2013). Our results suggest that handedness and manual specialization are associated with the later stage of motor planning and execution, which takes place in the SLF I.

### Frontoparietal Tract Asymmetry, Handedness and Manual Specialization

Our findings of a link between structural asymmetry of the SLF I, hand preference and manual specialization extend previous studies and suggest an anatomical mechanism for hand dominance. For individuals with right hand preference, leftward structural asymmetry of the SLF I may enable faster integration of right body proprioceptive and tactile information with visual percepts from the side of peripersonal space (i.e., the right hemifield) in which their dominant hand is more likely to operate (Verfaellie and Heilman 1990; Howard et al. 2009). The finding of an opposite correlation in left-handers suggests a similar but reversed mechanism for those with left-hand dominance. The volumetric diffusion measurements we used to produce streamlines and quantify structural asymmetries have been demonstrated to correlate with histological and electrophysiological properties of myelinating axons in the developing brain (Drobyshevsky et al. 2005). In addition, asymmetry of, specifically, tract volume can be a useful measure to assess behavioral asymmetries as previously demonstrated in studies of visuospatial attention tasks. Using a Posner Paradigm, we demonstrated that right-handed participants who responded faster to stimuli appearing in one hemifield had a larger volume in the contralateral SLF II (Thiebaut de Schotten, Dell’Acqua et al. 2011). Reaction time may depend on several neurobiological mechanisms including faster conduction velocity along tracts composed of more myelinated, larger axons. Hence, the volumetric asymmetry demonstrated in our results may reflect several neurophysiological mechanisms, which in turn may determine interhemispheric differences observed in fMRI activation (Begliomini et al. 2008) and differences in behavioural performance between the two hands. Similarly, the finding of an association between reduced manual specialization and a more symmetrical pattern of SLF I is in line with fMRI and MEG studies showing more symmetric hemispheric activation during motor tasks performed equally well with both hands (Dassonville et al. 1997; Volkmann et al. 1998). However, the cross-sectional design of our study and the correlative analysis prevent us from evaluating whether prolonged preference to use one hand over the other leads to the observed hemispheric asymmetry, or whether innate hemispheric differences in fact determine individual hand preference.

The results showing significant correlations between SLF I asymmetry and peg manipulation but not finger tapping suggest that manual specialization is directly linked to connections that mediate somatosensory and visuospatial integration and feedback. Considering that assessments of handedness are heavily based on self-report on object manipulation tasks, our findings support the hypothesis that hand preference and manual specialization share a common cognitive mechanism. This conclusion is further supported by previous studies that show differences exist in motor speed between right- and left-handers for tasks requiring visual feedback, and that visual feedback has more impact on performance than task difficulty (Todor and Doane 1978; Gonzalez et al. 2006). These differences could be related to an attentional bias toward the dominant arm, which in turn may lead to a faster speed to reach the required target (Honda 1982, 1984; Gardner and Potts 2010). Our results also support the theory that a hand-hemisphere dominance may exist, especially for movements requiring sensory feedback control (Flowers 1975). This was supported by our behavioral results in the Pegboard task, as participants performed much better with their dominant hand relative to the nondominant, when performing the place condition task that required a higher level of multisensory integration and visually guided feedback for appropriate sequencing of movement. The degree of difference between hands was much smaller in the remove condition where peg manipulation required less visually guided precision, a similar result to that observed in a larger study of 153 subjects (Bryden and Roy 2005; Albuquerque et al. 2017). Overall, the above considerations indicate that hand preference is a complex construct in which the nature of the task and the underlying cognitive mechanisms used to assess the motor performance is pivotal in revealing anatomical–behavioral correlations and in determining the direction of manual dominance.

Finally, while our results indicate a shared anatomical and possibly cognitive mechanism betwen hand preference and manual specialization, they also clearly showed the existence of a spectrum of lateralized performance on Pegboard tasks, with many participants performing equally well with both hands despite being classified as right- or left-handers by the EHI. This confirms that a correct scientific approach to handedness should not be based on simple behavioral dichotomies or explained by specular hemispheric dominance. Multivariate pattern analysis of fMRI data acquired in right-handers has demonstrated that regions connected by the SLF I code for both ipsilateral and contralateral upper limbs (Gallivan et al. 2013), with some specialization between the two hemispheres (Sainburg 2002; Gonzalez et al. 2006; Begliomini et al. 2008; Przybyla et al. 2012). For example, in right-handers the left hemisphere is thought to specialize for co-ordinating the dynamics of movement across multiple segments, and the right hemisphere has advantages in refining limb position for better accuracy (Sainburg 2002; Przybyla et al. 2012). Our results are against the interpretation of a reverse hemispheric dominance or specialization in left-handers. Indeed, the anatomical similarities in the left SLF I between right- and left-handers and similar performances with the right hand between the two groups may reflect a hard-wired left hemisphere dominance for precision grasping irrespective of the hand preference, as already suggested by previous behavioral studies both in humans (Gonzalez et al. 2006, 2007; Begliomini et al. 2008) and chimpanzees (Hopkins et al. 2002; Forrester et al. 2012). Conversely, the larger right SLF I and superior performance with the left hand observed in left-handers compared with right-handers suggest that the higher anatomical variability in the right hemisphere may carry a specific advantage for developing better manual skills with the left hand, but only in left-handers. A direct consequence of our findings is that the anatomical heterogeneity in the right hemisphere may be the key factor that explains interindividual variability in manual specialization and hand preference among the general population. While we are unable to determine the exact mechanisms associated with this right hemisphere heterogeneity, our hypothesis is relevant to current approaches to motor rehabilitation and offers a clear anatomo-functional mechanism that can be tested in future studies by combining tractography with fMRI and kinematic data.

### Asymmetry of Projection Tracts

Our study confirmed previous reports of a leftward hemispheric asymmetry of the corticospinal tract, but no effect of handedness (Kertesz and Geschwind 1971; Westerhausen et al. 2007; Seizeur et al. 2014). There was no pattern of lateralization for the superior cerebellar peduncle in both right- and left-handers, without a significant effect of handedness.

Previous studies examining structural asymmetry in the brain have identified an effect of handedness on the morphology of the grey matter where the majority of the corticospinal tract terminates, in the precentral gyrus. In particular, a recent large study in 250 individuals (120 left handers) showed an effect of handedness on sulcal depth in the Rolandic genu but no effect on asymmetry of surface area, cortical thickness or volume (Maingault et al. 2015). Their result showed a leftward depth asymmetry in right-handers and the converse in left-handers, a pattern echoed in previous studies examining brain morphology (Amunts et al. 1996, 2000; Foundas et al. 1998; Sun et al. 2012). One voxel-based morphometry (VBM) study of 112 participants (56 left-handers) has also reported differences in only the grey matter of precentral regions (Hervé et al. 2006) although two other VBM studies in larger cohorts of 465 participants (67 left handers; Good et al. 2001) and 142 participants (14 left-handers; Watkins et al. 2001) reported no effect of handedness. Another approach has been to analyse diffusion-weighted maps (fractional anisotropy), and this was performed in 28 volunteers (9 left-handed), reporting an effect of handedness on asymmetry of the FA under the precentral gyrus near the hand region (Büchel et al. 2004). The differences between our findings and previous voxel-wise approaches is not surprising considering that tractography measures structural properties along the whole tract bundle, and is limited in reaching the intracortical portion of the fibers. Previous reports of structural asymmetry of the precentral gyrus were also limited to describing differences between right- and left-handers, with few correlations with behavioral data reported. Hence, it remains to be demonstrated whether differences in the precentral gyrus reflect a direct effect of handedness on cortical anatomy or an indirect effect through its connections, possibly mediated by association and projection tracts that were not considered in our study (e.g., U-fibers or thalamic projections to precentral gyrus) (Catani 2017).

### Limitations and Conclusions

Some limitations of our study deserve further explanation. We were unable to test exactly which component of movement (online control or planning) was driving the observed association between the pegboard and tract asymmetry, which future kinematic studies may be able to elaborate (Budisavljevic, Dell’Acqua et al. 2016). A mixed sample of male and female participants was also used, and sex differences in manual performance and hemispheric asymmetry have been reported in some previous studies (Good et al. 2001; Bryden and Roy 2005). However, all our analyses were performed using sex as covariate and a lateralization index that compared the hands within-subject to minimize the possible effect of finger size and other factors in task performance (Peters and Campagnaro 1996; Bryden and Roy 2005).

Our sample size was not smaller than many other neuroanatomical studies of structural asymmetry of hand preference and left-hand behavioral performance (Dassonville et al. 1997; Foundas et al. 1998; Boulinguez et al. 2001; Büchel et al. 2004; Seizeur et al. 2014). However, we acknowledge that our findings need to be replicated using larger study groups (Mazoyer et al. 2016). It would also be important to assess whether different neural structures are important when using familiar (tools) versus nonfamiliar (pegs) objects to evaluate manual specialization (Bi et al. 2015) and extend the analysis to other tracts of the frontal and parietal lobe (Catani, Dell’Acqua et al. 2012; Catani et al. 2017). We were also unable to test for the larger spectrum of language dominance in the left-handers, a more heterogenous group as compared with right-handers also with regard to the degree of hand preference. As hemispheric dominance for praxis and language may co-lateralize, an assessment of this would have been useful to understand their mutual interaction. Finally, the cross-sectional nature of our study precludes making any conclusion on whether anatomical differences determine behavioral patterns, or are shaped by preferred usage of one hand over the other. Future longitudinal studies in early developmental stages or studies in twins or corrected left-handers may help to determine whether tract asymmetry is a precursor to manual specialization and hand preference, and clarify the effects of familial and environmental factors on the interaction between brain development, hand preference and social cognition (McManus 2002; Annett 2008; Budisavljevic et al. 2015; Budisavljevic, Kawadler et al. 2016).

In conclusion, our results suggest that handedness and manual specialization may have a common structural correlate in the dorsal frontoparietal system (SLF I). We showed an association between anatomical asymmetry of the volume of the SLF I and lateralized manual performance on a visually guided peg placing task, suggesting that integration of ipsilateral somatosensory and visual information is key to the development of lateralized motor behaviour in humans. Anatomical differences between hemispheres helps to predict manual specialization in humans and understand possible mechanisms that operate in the two hemispheres. We suggest that the lack of difference in the anatomy of the left SLF and right hand performance between left- and right-handers indicate a hard-wired left hemisphere dominance for movements with the right hand irrespective of the hand preference. A larger SLF I in the right hemisphere than the left hemisphere facilitates faster communication between regions involved in the planning and online control of the left arm and hand interacting with the external environment. This mechanism seems to operate only in the left-handers as indicated by their faster left hand performance speed on certain motor tasks compared with right-handers. Whilst the hands have the same potential abilities, the choice of one hand over another may be reflected in the speed of integration between association regions especially in the right hemisphere.

## Supplementary Material

Supplementary Data
