# Supplementary material for: Frontoparietal Tracts Linked to Lateralized Hand Preference and Manual Specialization
Source: Cereb Cortex. 2018 Apr 21;28(7):1–13. doi: 10.1093/cercor/bhy040 (PMC6005057; doi:10.1093/cercor/bhy040)
Supplement: Supplementary Data [file bhy040supplementarymaterial.docx]

**Supplementary Material**


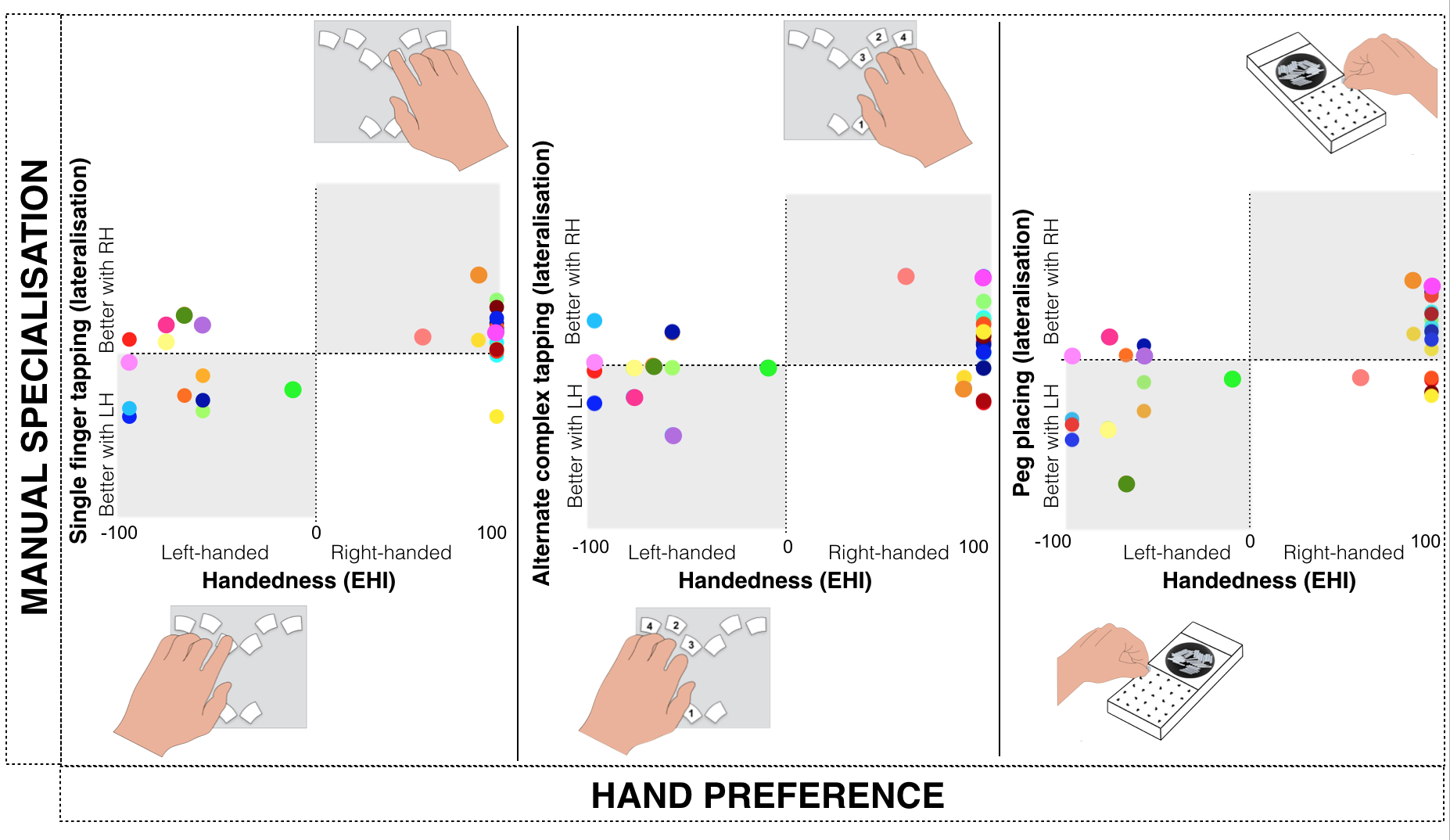


**Supplementary Figure 1.** Relationship between hand preference (shown as EHI scores) and manual specialization (shown as lateralization indices between hands) across the two tasks under two conditions. Colored dots represent the same subject.


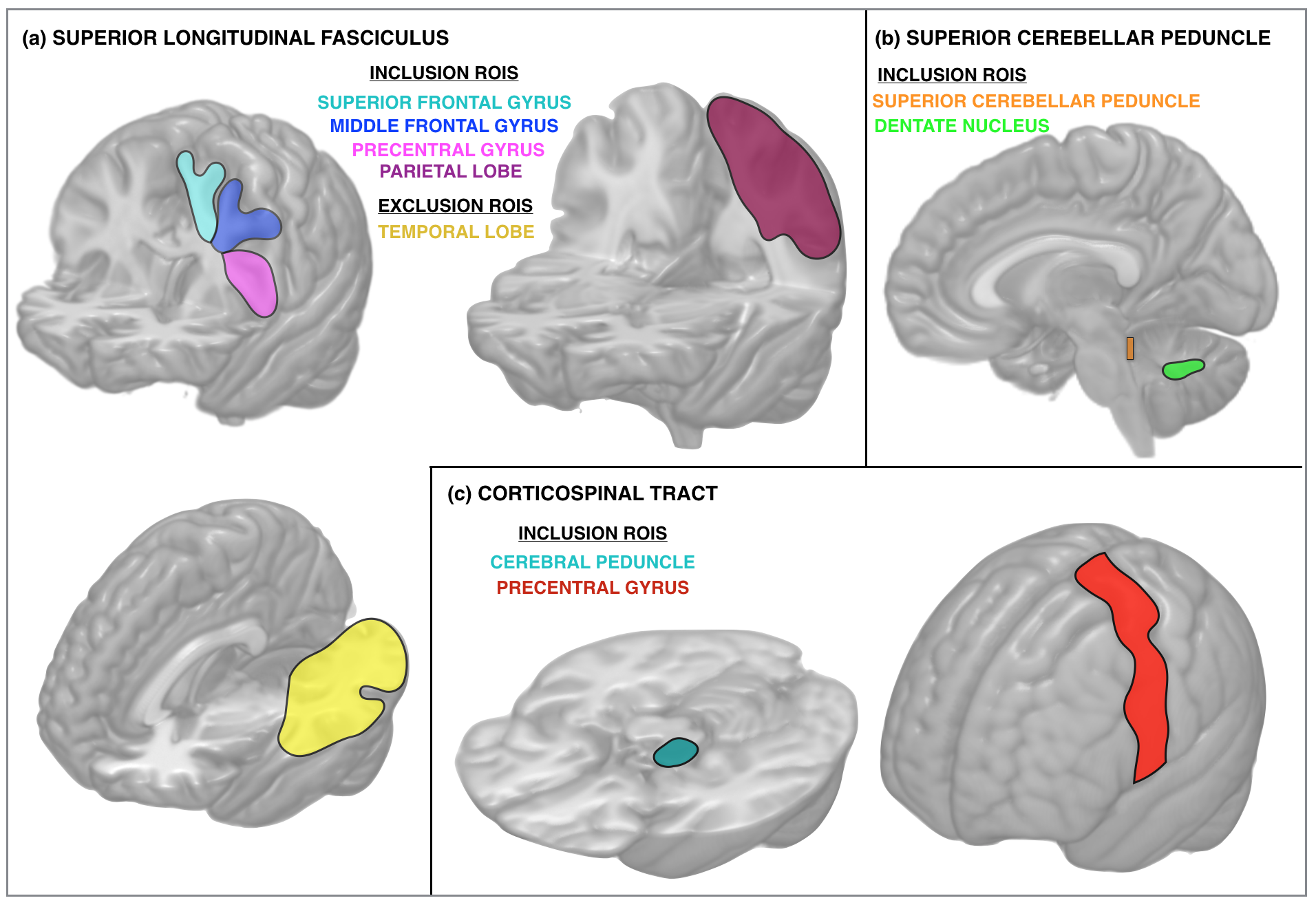


**Supplementary Figure 2.** Inclusion and exclusion regions of interest used in tractography dissection for (a) SLF I, II and III (b) superior cerebellar peduncle and (c) corticospinal tract

Supplementary Figure 2 shows a composite image of the cortical projections of the SLF I, II and III. The brain surface of the MNI152 T1 template was reconstructed using BrainSuite (Shattuck & Leahy, 2002). The end-points of each streamline from the dissected group tractogram were then used to determine the terminal projections on the white matter surface of the T1 template using Matlab. Briefly, the projection of each point was calculated by including any vertices of the white matter surface within a 3 mm radius from that point. This radius allowed most end-points to project within the corresponding gyrus without erroneously reaching adjacent gyri. The external pial surface was not used in this step because tractography streamlines often terminate within gyral white matter before reaching the cortex. A precise representation of the true projections is difficult due to the nature of tractography data but this method provides a good approximation nonetheless, especially at the gyral scale. The final visualization was performed in Surf Ice (<https://www.nitrc.org/projects/surfice/>).


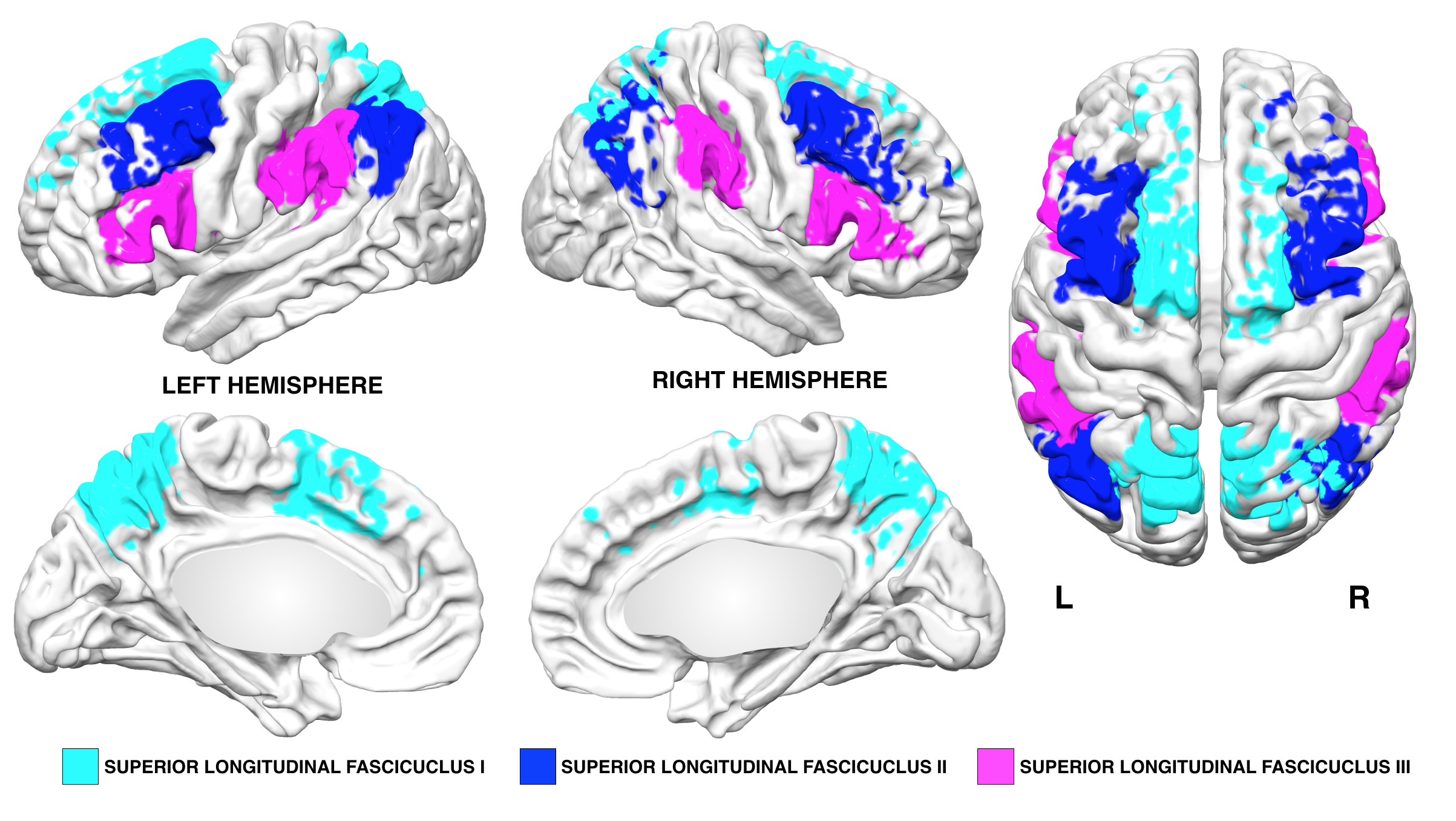


**Supplementary Figure 3.** Cortical projections of the SLF I, II and III in the left and right hemisphere in healthy adults, displayed on an MNI template brain.

**Supplementary Table 1.** Logistic regression model showing relationship between handedness and tract asymmetry (Lateralisation Index)

| **Tract Asymmetry** | **B** | **SE** | **Wald** | ***df*** | ***p*** | **Exp(B)** | **95% CI for Exp(B)** | |
| --- | --- | --- | --- | --- | --- | --- | --- | --- |
| **(Lateralisation Index)** |  |  |  |  |  |  | **Lower** | **Upper** |
| **SLF I** | -23.207 | 7.889 | 8.652 | 1 | 0.003 | 0.000 | 0.000 | 0.000 |
| **SLF II** | 5.992 | 3.193 | 3.440 | 1 | 0.064 | 373.274 | 0.715 | 194897.207 |
| **SLF III** | -6.979 | 4.676 | 2.227 | 1 | 0.136 | 0.001 | 0.000 | 8.907 |
| **Corticospinal tract** | 7.475 | 9.693 | 0.596 | 1 | 0.440 | 1763.576 | 0.000 | 3.078E+11 |
| **Superior cerebellar peduncle** | 3.797 | 4.439 | 0.732 | 1 | 0.392 | 44.554 | 0.008 | 267494.041 |

**Supplementary Table 2.** Logistic regression model showing relationship between handedness and tract asymmetry using different order on entry (Lateralisation Index)

| Tract Asymmetry (Lateralisation Index) | B | S.E | Wald | df | p | Exp(B) |
| --- | --- | --- | --- | --- | --- | --- |
| SLF III | -6.979 | 4.676 | 2.227 | 1 | .136 | .001 |
| SLF II | 5.922 | 3.193 | 3.440 | 1 | .064 | 373.274 |
| Corticospinal tract | 7.475 | 9.683 | .596 | 1 | .440 | 1763.576 |
| Superior cerebellar peduncle | 3.797 | 4.439 | .732 | 1 | .392 | 44.554 |
| SLF I | -23.207 | 7.889 | 8.652 | 1 | .003 | .000 |
|  |  |  |  |  |  |  |

**Additional Analysis:**

To exclude that a difference in degree of hand preference between the two groups, with a lesser extreme of handedness in the left-handed group, could affect the association observed, an analysis using only a homogenous subset of left-handers (EHI <-80) and right handers (EHI > 80) was performed. The differences between left- and right-handers for the right hand remained significant. This suggests that while the two groups are not equally distributed in terms of EHI (the left-handers being more heterogeneous) left-handers with EHI scores indicative of reduced laterality (e.g. EHI > -80) may not necessarily perform better with their right hand compared with the ‘strong’ left-handers (EHI -100).

**Supplementary Table 3.** Associations between dominant hand performance and tract volume

|  | **Dominant hand** | **Left hemisphere** | | | | | **Right hemisphere** | | | | |
| --- | --- | --- | --- | --- | --- | --- | --- | --- | --- | --- | --- |
|  |  | **SLF I** | **SLF II** | **SLF III** | **CST** | **SCP** | **SLF I** | **SLF II** | **SLF III** | **CST** | **SCP** |
| **Left handers** | **Peg place** | 0.167  0.624 | 0.551  0.79 | 0.070  0.839 | -0.558  0.074 | 0.481  0.134 | -0.157  0.644 | 0.247  0.464 | -0.445  0.170 | -0.461  0.154 | 0.197  0.562 |
|  | **Peg remove** | 0.395  0.229 | 0.339  0.307 | 0.313  0.349 | -0.183  0.590 | -0.136  0.690 | 0.336  0.313 | 0.081  0.813 | 0.455  0.160 | -0.329  0.323 | -0.327  0.326 |
|  | **Multifinger tap** | -0.202  0.551 | -0.266  0.429 | 0.070  0.839 | 0.195  0.565 | 0.015  0.965 | -0.473  0.142 | -0.348  0.295 | -0.490  0.126 | 0.112  0.744 | -0.297  0.374 |
|  | **Index tap** | -0.044  0.899 | 0.050  0.884 | 0.189  0.579 | 0.040  0.907 | -0.132  0.698 | 0.000  1.000 | 0.485  0.130 | 0.446  0.169 | -0.089  0.794 | -0.066  0.847 |
| **Right handers** | **Peg place** | -0.544  0.055 | **-0.587**  **0.035*** | -0.499  0.083 | 0.280  0.354 | -0.185  0.545 | -0.355  0.235 | -0.431  0.141 | -0.148  0.630 | 0.044  0.886 | 0.006  0.985 |
|  | **Peg remove** | -0.041  0.895 | **-0.624**  **0.023*** | -0.023  0.941 | -0.062  0.840 | -0.145  0.637 | 0.021  0.945 | **-0.594**  **0.032*** | -0.033  0.914 | -0.288  0.341 | 0.233  0.444 |
|  | **Multifinger tap** | 0.481  0.082 | 0.263  0.363 | **0.669**  **0.009*** | -0.166  0.572 | 0.513  0.061 | 0.555  0.071 | 0.167  0.569 | 0.048  0.870 | -0.237  0.414 | 0.463  0.096 |
|  | **Index tap** | -0.191  0.512 | 0.053  0.857 | 0.054  0.855 | 0.143  0.626 | 0.360  0.207 | -0.138  0.639 | -0.205  0.482 | 0.176  0.547 | -0.202  0.488 | 0.327  0.254 |

Note: Values are Pearson’s r (with significance levels *p<0.05). Pegboard values are average time (seconds) to completion therefore lower values indicate better performance, whereas finger tapping values are number of taps within 10 seconds over 5 trials and therefore higher values indicate better performance.
